# The role of simulation-based training in healthcare-associated infection (HAI) prevention

**DOI:** 10.1017/ash.2021.257

**Published:** 2022-02-07

**Authors:** Minji Kang, Madhuri B. Nagaraj, Krystle K. Campbell, Ian A. Nazareno, Daniel J. Scott, Doramarie Arocha, Julie B. Trivedi

**Affiliations:** 1 Division of Infectious Diseases and Geographic Medicine, Department of Medicine, University of Texas Southwestern Medical Center, Dallas, Texas; 2 Simulation Center, University of Texas Southwestern Medical Center, Dallas, Texas; 3 Infection Prevention, University of Texas Southwestern Medical Center, Dallas, Texas

## Abstract

**Objectives:**

To perform a review of the literature on the role of simulation-based training (SBT) in healthcare-associated infection (HAI) prevention and to highlight the importance of SBT as an educational tool in infection prevention.

**Methods:**

We reviewed English language publications from PubMed to select original articles that utilized SBT as the primary mode of education for infection prevention efforts in acute-care hospitals.

**Results:**

Overall, 27 publications utilized SBT as primary mode of education for HAI prevention in acute-care hospitals. Training included the following: hand hygiene in 3 studies (11%), standard precaution in 1 study (4%), disaster preparedness in 4 studies (15%), central-line–associated blood stream infection (CLABSI) prevention in 14 studies (52%), catheter-associated urinary tract infection (CAUTI) prevention in 2 studies (7%), surgical site infection prevention in 2 studies (7%), and ventilatory associated pneumonia prevention in 1 study (4%). SBT improved learner’s sense of competence and confidence, increased knowledge and compliance in infection prevention measures, decreased HAI rates, and reduced healthcare costs.

**Conclusion:**

SBT can function as a teaching tool in day-to-day infection prevention efforts as well as in disaster preparedness. SBT is underutilized in infection prevention but can serve as a crucial educational tool.

Simulation-based training (SBT) utilizes artificial representation of real-world processes to allow for learning with approximation of practice. It is an educational technique, not a technology, that facilitates learning through immersion, reflection, feedback, and practice in a controlled environment with minimal risk to patients. The Institute of Medicine’s “To err is human” highlighted medical errors and their consequences, emphasizing the need for patient safety through the design of a safer health system.^1^ Common root causes of preventable medical errors were communication breakdowns, faulty systems of care, lack of standardization in practice, and insufficient knowledge.^1,2^ By allowing practice in a realistic and interactive setting with minimal risk to patients, SBT can help minimize medical errors by acquiring clinical skills through practice, improving communication skills, defining team structures, and refining protocols.^3,4^

According to the Centers for Disease Control and Prevention (CDC), 1 in 31 hospitalized patients developed at least 1 healthcare-associated infection (HAI) in 2018.^5^ Most HAIs are related to invasive devices or procedures: catheter-associated urinary tract infection (CAUTI), central-line–associated bloodstream infection (CLABSI), surgical site infection (SSI), and ventilator-associated pneumonia (VAP). Evidence-based prevention strategies can reduce HAIs but are insufficiently implemented. Education is a key component of infection prevention efforts and traditional methods of teaching include lectures, videos, and fact sheets with some opportunities for hands-on practice.^6^ With growing recognition of SBT in healthcare education, SBT can also be used as an important adjunct to traditional teaching and assessment methods in infection prevention. In this study, we performed a focused literature review on the use of SBT in HAI prevention and highlight the importance of SBT as an educational tool in infection prevention.

## Methods

We reviewed English language publications from PubMed using combinations of keywords “simulation,” “infection prevention,” “healthcare-acquired infections,” and “disaster preparedness.” From this, we selected original articles that utilized SBT as the primary mode of education for infection prevention efforts in acute care hospitals. We defined simulation as the mode of training that utilized imitation or representation of one act or system by another. The references for each relevant paper were additionally reviewed.

## Results

We retrieved 138 English language publications from PubMed, of which 111 were excluded because simulation was not utilized for infection prevention education in acute-care hospitals. We then performed a detailed review of 27 publications that utilized simulation as primary mode of education for HAI prevention in acute care hospitals (Table 1).

**Table 1.** Simulation in Healthcare-Acquired Infection Prevention: Review of Medical Literature

Infection Prevention Measure	Trainer	Learner	Training	Outcome	Reference
**General**					
Hand Hygiene	Critical care physicians, hygienist nurses	Second- and third-year medical students	Application of fluorescent alcohol-based hand rub under UV-C	Improvement in complete application of alcohol hand rub	Dray et al^7^
	Does not specify	Residents, nurses, nursing assistants			Ghazali et al^8^
	Chief nurses	Healthcare workers			Lehotsky et al^9^
Standard precautions	Does not specify	Nursing students	Donning and doffing of PPE; performing nursing practices on standardized patients or peer role play	Increase in knowledge, awareness of standard precaution and infection control performance	Kim et al^10^
**Disaster/outbreak preparedness**					
PPE	Educator, director of infection control	Nurses, physicians, respiratory therapists	Cardiac arrest scenario in patient with SARS	Identified errors in infection control measures	Abrahamson et al^11^
	Does not specify	Physician, nurses, technicians	Deterioration of patient with suspected Ebola	Improvement in post-intervention score	Abualenain et al^12^
	Physicians	Multidisciplinary healthcare workers	Application of PPE; practice of various procedures on patient with Ebola	Increasing sense of security, predisposition, and confidence	Carvalho et al^13^
Hand Hygiene, PPE	Nursing champions, simulation team	Nurses, physicians, respiratory therapists	Tracking of surface contamination using UV-C luminescent spray during MRSA outbreak	No new episodes of colonization or infection	Gibbs et al^14^
**CAUTI**					
Urinary catheter Insertion	Nurse educator	Medical students	Aseptic technique during insertion of urinary catheter	Lowest CAUTI rate among medical students	Barnum et al^15^
	Simulation fellow, senior general surgery residents	Second year medical students	Simulated germs for hand washing, maintenance of aseptic technique during urinary catheterization	Maintained better sterility and had higher technical proficiency score during urinary catheterization	Mittal et al^16^
**CLABSI**					
Central venous catheter insertion	Vascular access nurse or physician	Attending physicians, residents	Hands-on practice of insertion of central venous catheter	Decrease in CLABSI rate	Allen et al^17^
	Critical care fellows, attending physician	Residents			Burden et al^18^
	Neonatologists	Attending physicians, residents			Steiner et al^19^
	Does not specify	Internal medicine emergency medicine residents		Fewer CLABSI after intervention Increased cost savings	Barsuk et al^20^ Cohen et al^21^
	Does not specify	Residents in anesthesia		Improvement in compliance on catheter insertion checklist	Cartier et al^22^
	Emergency medicine and critical care attendings	Emergency medicine residents		Improvement in sterile technique performance scores	Hoskote et al^23^
	Does not specify	Second- and third-year medical residents		Improvement in sterile technique score	Khouli et al^24^
	Anesthesiologist, Pulmonary/critical care physician	Interns, residents, nurse anesthetists		Increase in Likert-scale ratings on aseptic technique	Latif et al^25^
	Infection control practitioners, hospital epidemiologist	Medical students, Interns		Increase use of full-size sterile drapes, decrease in rate of catheter-related infection, cost savings	Sherertz et al^26^
Central venous catheter maintenance	Nurses	Student nurses	Hands-on practice of maintenance of central venous catheter	No difference in postest score for lecture-based vs simulation based	Aloush et al^27^
	Investigator	Nurses		Improvement in compliance bundle score	Hebbar et al^28^
	Nurses	Parents of children with cancer		Difference in knowledge score pre-and post-test	Rosenberg et al^29^
	Clinical educators	Nurses		Decrease in CLABSI rate	Scholtz et al^30^
**SSI**					
Surgical hand disinfection, preparation of surgical field	Surgical nurse, infection preventionist	Medical students, operating room technician trainees	Hands-on practice of operating room entry procedure, surgical hand disinfection, skin preparation	High satisfaction among learners	Breckwoldt et al^31^
Surgical hand rub technique	Does not specify	Medical students	Fluorescent solution, hands placed under UV-C light	Improvement in compliance and efficacy of surgical hand rub	Vanylos et al^32^
**VAP**					
Oral care	Does not specify	Critical care nurses	Hands-on practice of ventilator bundle related to oral care practices	Increase in knowledge score	Jansson et al^33^

Note. UV-C, ultraviolet C; PPE, personal protective equipment; SARS, severe-acute respiratory syndrome; MRSA, methicillin-resistant *Staphylococcus aureus*; CAUTI, catheter-associated urinary tract infection; CLABSI, central-line–associated blossdstream infection; SSI, surgical site infection; VAP, ventilator acquired pneumonia.

Trainers included the following: physicians in 8 studies (30%), nurses in 7 studies (26%), infection preventionists in 3 studies (11%), and simulation center staff in 2 studies (7%). Learners included the following: residents in 10 studies (37%), nurses in 9 studies (33%), medical students in 7 studies (26%), and physicians in 5 studies (19%). Nursing students, technicians, and respiratory therapists made up a minority of learner types.

Furthermore, 3 studies focused on SBT for hand hygiene using the application of fluorescent alcohol-based hand rub under ultraviolet C light with all studies demonstrating improvement in complete application of alcohol hand rub.^7–9^ In addition, 3 studies used SBT to recreate clinical scenarios pertaining to suspected Ebola and severe acute respiratory syndrome (SARS) cases^11–13^ with identification of errors in infection control measures,^11^ and improvement in both postintervention scores^12^ and learners’ sense of confidence.^13^ Also, 13 studies utilized SBT for CLABSI prevention, and 10 studies focused on aseptic technique pertaining to central venous catheter (CVC) insertion^17–26^ with improvement in sterile technique^20–27^ and CLABSI rate.^17–19^ We identified 4 studies that focused on CVC maintenance^27–30^ with improvements in compliance in bundle usage^28^ and decrease in CLABSI rates.^30^ We identified 2 studies that utilized SBT for Foley catheter insertion^15,16^ with improvement in sterile technique^16^ and decreased CAUTI rates.^15^ Finally, 2 studies focused on SSI prevention with surgical hand washing technique and preparation of the surgical field.^31,32^

## Discussion

SBT is underutilized in infection prevention, but it can serve as an important adjunct to traditional educational tools. It can target learners ranging from students to nurses, physicians, and other ancillary staff. SBT programs in infection prevention can vary widely from disaster preparedness of high acuity to low-frequency clinical scenarios to day-to-day infection prevention measures. SBT can improve learner’s sense of competence and confidence,^11–13^ increase patient safety through improved compliance in infection prevention measures^16,22–26,28,32^ and increase in knowledge,^29,33^ improve HAI rates,^15,17–20^ and reduce healthcare costs.^21^

Simulation modalities can offer a realistic imitation under test conditions. Different simulation modalities and environments are classified as low fidelity, medium fidelity, and high fidelity, with the highest fidelity modality or environment most accurately representing the real environment. Various simulation modalities have been integrated into infection prevention efforts, each with its own advantages and disadvantages (Table 2). Task trainers (low-fidelity simulators) have been predominantly utilized to help learners practice specific psychomotor skills such as aseptic technique in the insertion of central venous catheters^17–26^ and indwelling urinary catheters.^15,16^ Standardized patients, which are real people portraying the role of patients, have been utilized to practice standard precautions and interpersonal skills.^10^ Virtual reality (high fidelity) has also taught healthcare personnel to safely don and doff PPE,^34^ as virtual reality help learners to gain knowledge on the proper process of a procedure in a scalable fashion. In prior outbreaks, mid- and high-fidelity manikins, which are full-body manikins, were utilized to mimic patient encounters with rare communicable diseases like Ebola and SARS, aiding in the identification of errors in infection control measures.^11–13^ These simulators can replicate a patient’s physiology modeling through the programming of vitals, breathing, or other patient presentations. They enable a team to work collectively to deliver care (eg, intensive care unit setting with in full hazmat suit) while performing a certain set of procedural tasks (eg, intubation) in a physical environment. This enables teams to practice not only individual skills, but also critical teamwork and communication skills.

**Table 2.** Simulation Modalities

Modality	Description	Advantage	Disadvantage
Computerized virtual patients	Computer technology to create an on-screen virtual patient or scenario	Able to accommodate large groupsKeep students engaged	Difficult to develop communication or procedural skills
Task trainers	Device used to simulate a specific task, procedure, or skill	Lower in cost	Difficult to accommodate large groups
		Ideal for specific task that require repeated practice	
Standardized patients	Human actors hired as a role player	Able to communicate in realistic manner and develop communication skills	Unable to simulate high risk or invasive procedures
			Difficult to use in large groups
Mid-fidelity manikins	Full body simulated patient with minimal computer components	Lower in cost compared to high fidelity options	Difficult to use in large groups
		Able to increase complexity of scenarios	Challenging for complex simulation
		Portable	Lack of verbal responses and difficult to develop communication skills
High-fidelity manikin	Full body computer-based simulated patient with ability to mimic	Able to speak and simulate physical exam findings	Expensive
		Drug recognition and response	Time intensive
		Cardiac monitoring capability	Technology dependent
		Wireless and somewhat portable	Difficult to use in large groups
Virtual reality	Interaction with a synthetic environment that exist solely in the computer	Enhanced visualization	Expensive
			Lack of human interaction
			Lacks flexibility

Ultimately, simulation experts match the learning objectives to the most effective modality. For effective learning, SBT should be offered as part of a curriculum to supplement and complement other educational methods with clearly defined objectives and benchmarks for learners to achieve. The program should provide individualized and team learning and should allow for repetitive practice with clinical variation such as varying levels of difficulty if feasible. Expert trained facilitators should provide feedback during or after a learning experience through rapid cycle deliberate practice or debriefing, based on learning objectives.^35^

SBT in infection prevention has predominantly focused on central venous catheter insertion and maintenance with various studies reporting significant improvement in compliance with sterile techniques and decrease in CLABSI rate.^17–30^ However, despite its proven effectiveness in CLABSI prevention, SBT has remained underutilized in other realms of infection prevention. Through repetitive and deliberate practice to develop procedural competence and immediate constructive debriefing by an expertly trained debriefer, SBT can allow for standardization of routine infection prevention measures and proper implementation of evidence-based prevention strategies to reduce HAI rates. In addition, SBT can play a role in understanding and optimizing workflows and bottlenecks in high-acuity, low-frequency encounters for disaster preparedness. Centralized SBT that promotes competency-based education can yield a large return on investment by reducing overall healthcare costs through improved quality of care, reduced HAI penalties, mitigation of readmissions, and decreased lengths of stay.^21^ This study demonstrates the benefits of SBT in an expanded role in infection prevention teaching and argues for continued innovation.

In summary, SBT is underutilized in infection prevention but can serve as a crucial educational tool. The coronavirus disease 2019 (COVID-19) pandemic has exposed our inadequacy in infection prevention training as healthcare personnel train on the job in high-risk clinical environments. SBT can bridge this gap and improve compliance with infection prevention measures through repeated education in standardized teaching sessions in a safe environment by reinforcing best practices and personal accountability.
